# Mobile Application-Based Interventions for People with Heart Failure: A Systematic Review and Meta-Analysis

**DOI:** 10.1155/2024/6859795

**Published:** 2024-07-29

**Authors:** Yun-Xia Ni, Xue-Hui Liu, Li He, Ya Wen, Gui-Ying You

**Affiliations:** Department of Cardiology West China Hospital Sichuan University/West China School of Nursing Sichuan University, Chengdu, China

## Abstract

**Aim:**

To examine the effectiveness of mobile health application-based interventions on mortality, hospitalization rate, self-care, and quality of life in people with heart failure.

**Background:**

Mobile health application-based interventions are reported to potentially help people with heart failure improve health-related clinical outcomes. However, evidence on the effects of mobile health application-based interventions on mortality, hospitalization, self-care, and quality of life remains inconclusive and limited.

**Methods:**

A systematic literature search was conducted in six databases (MEDLINE, CINAHL Plus with Full Text, PsycINFO, Web of Science, EMBASE, and CENTRAL) to identify relevant studies from inception to 21 October 2023. Two authors independently extracted the data and assessed the risk of bias using the Cochrane risk-of-bias tool. The meta-analysis was conducted in Review Manager (version 5.4) and the statistical software R 4.3.3. Sensitivity analysis and subgroup analysis were also performed. The certainty of the evidence was evaluated by the GRADE approach.

**Results:**

Twenty-four studies involving 2886 participants were identified in this review. The pooled analysis showed that mobile health application-based interventions had statistically significant beneficial effects on reducing heart failure-related hospitalization (RR = 0.72, 95% CI 0.57 to 0.91, *p*=0.01) and improving quality of life (SMD = 0.46, 95% CI 0.09 to 0.83, *p*=0.02), but had no statistically significant effects on all-cause mortality (RR = 0.90, 95% CI 0.66 to 1.25, *p*=0.47), cardiovascular mortality (RR = 0.87, 95% CI 0.59 to 1.26, *p*=0.24), all-cause hospitalization (RR = 0.74, 95% CI 0.39 to 1.42, *p*=0.29), or self-care (MD = −2.42, 95% CI −15.07 to 10.24, *p*=0.64). Subgroup analyses indicated that intervention duration and monitoring frequency may influence the effects of mobile health application-based interventions on quality of life.

**Conclusions:**

Mobile health application-based interventions were effective at reducing heart failure-related hospitalization and improving quality of life in people with heart failure. More well-designed randomized controlled trials are needed to strengthen the evidence. *Implications for Nursing Management*. Mobile health application-based interventions may have benefits for improving heart failure-related hospitalization and quality of life. More rigorous studies are warranted to confirm the effects of mobile health application-based interventions for people with heart failure.

## 1. Introduction

Heart failure is considered a global health care problem since it is associated with a high hospitalization rate, increased mortality, and impaired quality of life, leading to a heavy economic burden on global health care systems [1, 2]. Current guidelines suggest that continuous medical therapy and patient self-care play a core role in improving the prognosis of heart failure [3]. Self-care in heart failure refers to the specific health behaviors that patients perform to manage their disease and promote health [4]; these behaviors typically include daily weight monitoring, symptom recognition, medication adherence, lifestyle changes, and regular follow-up [3]. Appropriate treatment adherence and optimal self-care have positive effects on patients' outcomes (e.g., decreased mortality and improved quality of life) [5, 6]. Despite the proven benefits of self-care, people with heart failure exhibit poor self-care behaviors [7–9]. Only 42% of patients persistently undertake good self-care behavior [9]. Studies have shown that unmet needs for disease knowledge and limited access to healthcare providers are the main barriers to self-care for people with heart failure [10, 11].

Recent advances in information technology have offered opportunities to address and resolve self-care barriers for people with heart failure. Mobile health (mHealth) is defined as the use of mobile devices (e.g., smartphones, tablets, personal digital assistants—PDAs, and other mobile devices) to deliver health care services outside of hospital settings [12]. Mobile application is one of the typical and popular delivery methods for mHealth worldwide [13]. Interventions based on mobile health applications have the potential to educate one on one at any convenient time and place, collect real-time data, track heart failure signs and symptoms, and provide quick health advice [14, 15]. Previous trials demonstrated that mobile applications can promote patient adherence to medication, self-monitoring of symptoms, and healthy lifestyles, which contribute to lower rehospitalization rates and improved quality of life [15–17]. Although the potential of mobile health application-based interventions has been well established in recent years, the effectiveness of such interventions for people with heart failure is mixed [18–20]. For example, Jiang et al. [17] reported that smartphone application use could improve self-care and quality of life among people with heart failure, while no effect on self-care was found in another article [20].

Several systematic reviews have assessed the effects of mobile health application-based interventions on people with heart failure. Kitsiou et al. [21] revealed that mobile health interventions (such as mobile applications, text messages, and remote mobile monitoring) were effective at reducing mortality and heart failure-related hospitalizations. Son et al. [22] reported that mobile phone-based interventions, including voice calls, telemonitoring, short messaging services, and mobile applications, could reduce the length of hospital stay for people with heart failure. However, these reviews combined various mHealth interventions in one study instead of focusing on specific application-based interventions [21–23], which may confound the true effects of application-based interventions. One review tested the effect of mobile applications, but the participants were mixed patients with various cardiovascular diseases (i.e., hypertension, heart failure, stroke, and cardiac rehabilitation populations) and not categorizing for heart failure [24]. Athilingam and Jenkins [25] qualitatively synthesized the effects of mobile phone application interventions and found inconsistent effects on self-care management. In their review, the randomized controlled trials (RCTs), pilot RCTs, and prepost intervention design trials were included. One recent systematic review examined the effectiveness of mobile health applications on mortality, hospitalization rate, and quality of life, but the applications were limited to telemonitoring applications, and applications with no remote monitoring functionality were not included [26]. Additionally, this review did not assess the effects on self-care and only included articles published between 2000 and 2021. Alves Leite de Barros et al. [13] conducted a review on the effects of mobile health applications on people, focusing on medication adherence, but the authors did not evaluate mortality, hospitalization, self-care, or quality of life. Thus, existing reviews cannot provide sufficient evidence on the effects of mobile health application-based interventions on mortality, hospitalization, self-care, and quality of life in people with heart failure. Moreover, as the field of information technology has advanced, there has been rapid growth in mobile application intervention research. There shall be more rigorous data synthesis to inform the effects of mobile health application-based interventions to guide heart failure management. Therefore, this systematic review aims to quantitatively analyze the evidence of mobile health application-based interventions to determine its pooled effects on mortality, hospitalization rate, self-care, and quality of life for people with heart failure.

## 2. Methods

This systematic review and meta-analysis were registered in the International Prospective Register of Systematic Reviews (PROSPERO protocol number: CRD42023492005) and reported according to the Preferred Reporting Items for Systematic Reviews and Meta-Analyses (PRISMA) guidelines. A Measurement Tool to Assess Systematic Review 2 (AMSTAR2) was followed to ensure the quality of this review [27].

### 2.1. Literature Search

A comprehensive search was performed from inception to 21 October 2023 across six electronic databases: MEDLINE, CINAHL Plus with Full Text, PsycINFO, Web of Science, EMBASE, and CENTRAL. The search strategy used a combination of medical subject headings (MeSH) and text words, such as “heart failure,” “cardiac failure,” “mobile applications,” “mobile health,” and “smartphone.” Appropriate Boolean operators were used to combine these MeSH terms and text words to retrieve all relevant studies. The limiters were English language articles. A manual search through the reference lists of the included articles and previously published relevant reviews was also conducted to retrieve any additional studies. The detailed search strategy is presented in Supplementary Material Table S1.

### 2.2. Inclusion and Exclusion Criteria

The articles were included if they met the following PICOS criteria: (1) population: adults diagnosed with heart failure; (2) intervention: any mobile health applications used either as a standalone or combined with other delivery modes, such as telephone calls, text messages, or telemonitoring. The applications offer an interface through any kind of mobile device, for example, a smartphone, tablet computer, or PDA; (3) comparison: usual care, standard care, routine care, waitlist control or health education without mobile application; (4) studies including at least one of the following outcomes: mortality, hospitalization rate, self-care, or quality of life; and (5) study design: RCTs or pilot RCTs.

The exclusion criteria were as follows: (1) mixed participants with various chronic diseases or general cardiac disorders (e.g., hypertension, coronary heart disease, and atrial fibrillation) but not categorizing for heart failure; (2) primary intervention were telephone calls, videoconferences, e-mail, online social networking, or any other mobile intervention without applications; (3) comparisons were made between different applications in both the intervention group and control group; (4) applications as a control group compared to other mobile technology-based interventions, such as automated telemonitoring devices; (5) incomplete studies, such as study protocols or incomplete data; and (6) conference abstracts, book chapter reviews, or editorials.

### 2.3. Study Selection and Data Extraction

The results of the literature search were exported to EndNote ×9 software. After removing duplicates, two authors independently screened the titles and abstracts of the studies based on the inclusion and exclusion criteria. Then, full-text assessment of potentially relevant studies was conducted by the same two authors to select the eligible studies to be included in this review. Any disagreements were discussed with a third reviewer to reach a consensus.

Data from the included studies were independently extracted by two authors using predesigned data collection forms guided by the Cochrane Handbook [28]. The extracted data included author, year, country, sample size, sample characteristics (e.g., age, gender), intervention characteristics (e.g., key component, frequency and duration, mobile device, and delivery personnel), control characteristics, outcomes and measurement tools, and grants. The corresponding authors were contacted by e-mail for missing information when the data were incomplete. Disagreements were resolved by discussion with a third reviewer.

### 2.4. Quality and Evidence Assessment

Two authors assessed the methodological quality of the included studies using the Cochrane Risk-of-Bias Tool [29]. Disagreements were resolved by discussion with a third reviewer. This risk tool includes seven domains: random sequence generation (selection bias), allocation concealment (selection bias), blinding of participants and personnel (performance bias), blinding of outcome assessment (detection bias), incomplete outcome data (attrition bias), selective reporting (reporting bias), and other bias. Each domain was rated as having a low risk of bias, a high risk of bias, or an unclear risk of bias. The certainty of evidence was independently evaluated by two reviewers using the Grading of Recommendations Assessment, Development, and Evaluation (GRADE) approach [30], which classified the certainty of the evidence into four classes (high, moderate, low, or very low) from five aspects (risk of bias, inconsistency, indirectness, imprecision, and publication bias). The Grading of Recommendations Assessment, Development, and Evaluation Profiler Guideline Development Tool (GRADEpro GDT) was applied to develop the GRADE evidence profile.

### 2.5. Data Synthesis and Analysis

A descriptive synthesis was performed to describe the characteristics of the included studies. All the statistical analyses for the meta-analysis were performed with Review Manager 5.4 and the statistical software R 4.3.3. Binary outcomes were calculated by the risk ratio (RR) with 95% confidence intervals (CIs) using the Mantel–Haenszel method, and statistically significant RRs were translated into numbers needed to treat (NNT) to determine clinical relevance. For continuous data measured using the same tools, mean differences (MDs) with 95% CIs were used to determine the effects of the intervention. For continuous data measured using different tools, standardized mean differences (SMDs) with 95% CIs were computed using the generic inverse variance method [28]. The effect size was evaluated by Cohen's d (0.20–0.49 indicated a small effect, 0.50–0.79 a moderate effect and ≥0.80 a large effect) [31]. The overall effect was determined using Z-statistics with *p* < 0.05.

A random-effects model with restricted maximum likelihood estimation was used based on the clinical and methodological heterogeneity across the included studies [32, 33]. The Knapp–Hartung adjustments were conducted for 95% CIs of pooled effect sizes to reduce the risk of false positives. The 95% prediction intervals (PIs) were also calculated [34]. The statistical heterogeneity of the included studies was assessed using the *I*^2^ statistic and Cochran's *Q* test. *I*^2^ values of 0% to 40%, 30% to 60%, 50% to 90%, or 75% to 100% indicated unimportant, moderate, substantial, or considerable heterogeneity, respectively [28]. Possible sources of heterogeneity between studies were explored by subgroup analyses (i.e., duration of intervention, mobile device, frequency of monitoring, and delivery personnel). For studies with a multi-arm profile, the relevance to this review scope was assessed to create a single pairwise comparison for the main analysis according to the Cochrane Handbook [28]. When the studies reported the medians and interquartile ranges, the means and standard deviations were estimated following the recommendations of McGrath et al. [35]. A sensitivity analysis was performed by excluding one study at a time to test the robustness and reliability of the pooled results [36]. Egger's regression test and funnel plots were used to detect potential publication bias.

## 3. Results

### 3.1. Study Selection

**Figure 1:**
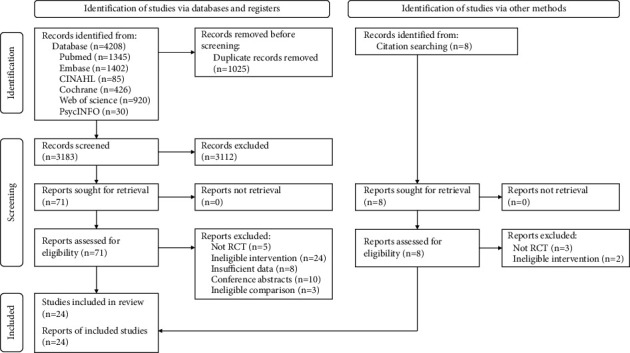
PRISMA flow diagram.

The PRISMA flowchart of the literature search and selection process is shown in Figure 1. A total of 4208 records were retrieved from electronic databases, and 8 records were searched manually through the reference lists of the included articles and previously published relevant reviews. After removing duplicates and screening for titles and abstracts, 79 studies remained for full-text assessment. Finally, 24 studies met the inclusion criteria and were included in this review.

### 3.2. Characteristics of the Included Studies

The characteristics of the included studies are shown in the Supplementary Material Table S2. A total of 2886 individuals with heart failure were involved from 24 studies, with sample sizes ranging from 18 to 710. Twenty-three studies [14–16, 18–20, 37–53] were two-arm trials, and one was a three-arm trial [17]. All the studies were published between 2009 and 2023, and 62.5% (15/24) of the studies were published after 2020. The studies were conducted in Europe (*n* = 10), North America (*n* = 6), Asia (*n* = 6), Oceania (*n* = 1), and South America (*n* = 1). The mean age of the participants ranged from 50.0 to 79.9 years, and the percentage of female participants ranged from 7% to 69.8%. Twenty-three studies involving 2855 participants reported the New York Heart Association (NYHA) classification (a classification system used to assess functional exercise capacity and symptom severity of patients), and approximately 50% presented with NYHA class III or IV. Left ventricle ejection fraction (LVEF) was described in 19 studies, 13 of which reported a reduced ejection fraction ≤40%.

### 3.3. Characteristics of Interventions and Comparators

Five studies delivered interventions via basic mobile phones, 11 studies via smartphones, 6 studies via tablets, and 2 studies via the PDA. All the studies conducted the interventions in participants' homes. The interventions were implemented by different personnel, including physicians, clinicians, cardiologists, or geriatricians, in 8 studies; nurses in 8 trials; multidisciplinary care teams in 5 studies; and study coordinators in 1 study. Two studies did not report any information about the personnel.

#### 3.3.1. Key Features of the Mobile Application-Based Interventions

The functionalities provided by the applications varied among studies. The seven key features of the applications included (1) reminders and notifications (i.e., reminding the participants to use the application, perform self-monitoring, or take medicine); (2) self-monitoring and assessment (i.e., conducting self-monitoring and recording vital parameters and symptoms, such as blood pressure, heart rate, and weight); (3) goal-setting (i.e., setting up a tailored goal for participants, such as physical activity); (4) health education (i.e., providing health information or resources related to heart failure); (5) feedback and alerts (i.e., providing clinician feedback and alerts based on physiological and symptom information); (6) tele-coaching (i.e., providing personalized recommendations and modifying the treatment); and (7) social interaction (i.e., access to chat rooms, discussion forums, and interactions with companions and professionals). The details of the interventions are summarized in the Supplementary Material Table S2.

*Reminders and notifications*: fifteen studies used applications that offered reminders and notifications. Visual or audible reminders or push notifications were sent to patients to remind them to take measurements (e.g., weight and blood pressure), answer the questionnaire (e.g., symptom assessment), or use the application.

*Self-monitoring and assessment*: all but one study [19] used applications with self-monitoring and assessment features. In 13 of these studies, physiological data (e.g., weight and heart rate) were automatically sent to mobile devices via wireless Bluetooth or wearable technology. Seven studies recorded the measurement values manually. Three studies did not report whether the measurements were manually recorded or automatically recorded. The most frequently monitored parameters across studies were weight (*n* = 20), blood pressure (*n* = 13), heart failure symptoms (*n* = 12), and heart rate (*n* = 7).

*Goal-setting*: three studies used applications that had goal-setting. Saleh et al. [15] set up a weekly goal for physical activity via the mobile health applications. Clays et al. [48] and Wita et al. [16] developed personalized physical exercise schemes or individualized care plans based on patient parameter trends.

*Health education*: thirteen studies used applications that offered health information and resources in various formats (e.g., text, photos, videos, question, and answer games). Health information involves various aspects of heart failure, such as pathophysiology, symptoms, symptom management, lifestyle, treatment, and self-care behavior.

*Feedback and alerts*: twenty studies used applications with feedback and alert features. Participants received feedback and alerts via e-mail, messages, calls or screen color classification (e.g., Red Zone) [44] when the reported vital parameters were outside the target range or when symptoms indicated possible worsening of heart failure. Feedbacks or alerts were generated based on medical staff assessments (delayed) [46, 50] or automatically generated by machines (immediate) [20, 41].

*Tele-coaching*: eight studies used applications with a tele-coaching feature. The tele-coaching included a clinical consultation, therapy modification (e.g., an extra dose of diuretic), an early office visit, and exercise prescription adjustment.

*Social interaction*: four studies used applications that allowed interactions. Among these studies, one study allowed patients to share their behavioral progression and daily goal achievement with their companions [15], and all four studies provided chat rooms or discussion forums for interactions between patients and professionals.

#### 3.3.2. Dosage (Duration and Frequency) of Mobile Application-Based Interventions

The duration of the mobile health application-based interventions varied among the included studies, ranging from 4 weeks to 24 months. Seven studies had the duration of mobile application-based interventions for 3 months. Six studies implemented the mobile application-based interventions for 6 months. Two studies reported the duration of the interventions for 12 weeks, two for 12 months, and two for 24 months. The remaining studies had the duration of mobile application-based interventions for 4 weeks, 6 weeks, 45 days, 8 weeks, or 240 days. The follow-up period ranged from 30 days to 2 years. Nineteen studies had daily self-monitoring for blood pressure, heart rate, weight, or symptoms. Three studies had weekly monitoring for the ECG. Twelve studies provided real time feedback or alerts. Further details are shown in the Supplementary Material Table S2.

#### 3.3.3. Empowerment Strategies

Eight studies [14, 15, 17, 19, 20, 44, 46, 51] incorporated empowerment strategies and behavior change strategies in the applications. Cichosz et al. [46] reported that the core concept of the intervention was patient empowerment, achieved by increasing patients' coping capabilities through self-monitoring. Saleh et al. [15] used the Theory of Planned Behavior to guide interventions and empower patients with the ability to perform physical activity. Johnson et al. [51], Athilingam et al. [44], and Kiyarosta et al. [14] reported that daily prompts or encouragement messages were sent to the patients in the intervention group. Another three studies [17, 19, 20] demonstrated that patients were motivated to engage in self-management behavior through various types of education information or daily messages. One study [48] embedded psychological support in applications, which included cognitive behavioral interventions and mindfulness exercises based on a weekly plan.

The control conditions, including usual care (*n* = 17), standard care (*n* = 6), and waitlist control (*n* = 1), varied across the included studies. The contents of the controls mainly included standard pharmacological treatment, provision of information, and outpatient follow-up.

### 3.4. Characteristics of the Study Outcomes

Study outcomes were measured using validated scales. Self-care was measured using two different scales, including the Self-Care of Heart Failure Index (SCHFI) and European Heart Failure Self-care Behavior Scale (EHFScB). Quality of life was measured using the Minnesota Living with Heart Failure Questionnaire (MLHFQ), Kansas City Cardiomyopathy Questionnaire (KCCQ), 36-item Short-form Health Survey (SF-36), or EuroQoL Five-Dimension. Nineteen studies reported outcomes of self-care or quality of life at baseline and follow-up, and one study provided change data from baseline to follow-up [48].

### 3.5. Risk of Bias and Certainty of Evidence

**Figure 2:**
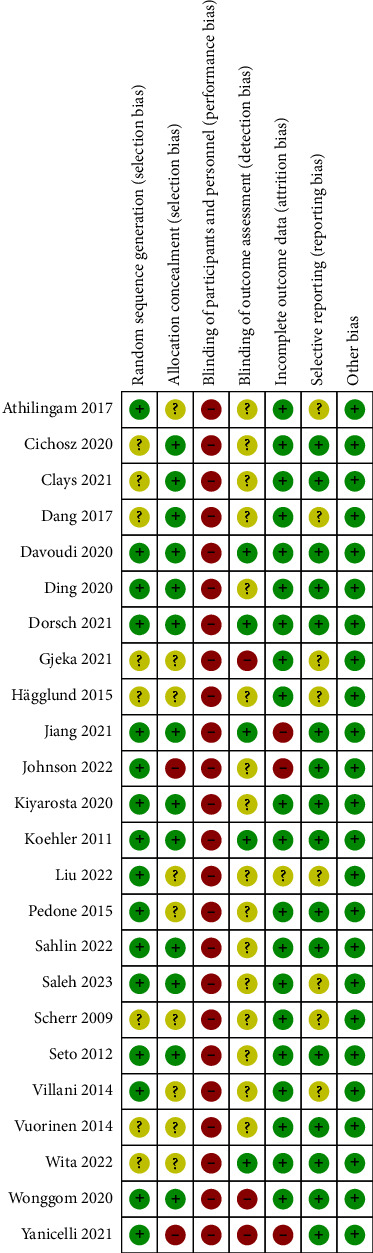
Risk of bias summary.

The risk of bias assessment for the 24 studies is presented in Figure 2. Sixteen studies provided details about random sequence generation, and 13 studies provided detailed information regarding allocation concealment, indicating a low risk of selection bias. Due to the nature of the mobile health application-based intervention, it was not feasible to blind the participants and personnels; thus, all studies were rated as high risk for performance bias. Blinding of outcome assessors was not achieved in 3 studies and was not clearly described in 16 studies, leading to a high or unclear risk of detection bias. Three studies were considered to be at high risk for attrition bias because the dropout rate was more than 20%, and intention-to-treat analyses were not performed. Eight studies were considered to have an unclear risk of reporting bias due to the lack of a published or registered protocol. The certainty of the evidence ranged from very low to moderate for different outcomes (Supplementary Material Table S3). The degrading factors mainly originated from the risk of bias, considerable heterogeneity, and small sample size.

### 3.6. Intervention Effects

#### 3.6.1. All-Cause Mortality

**Figure 3:**
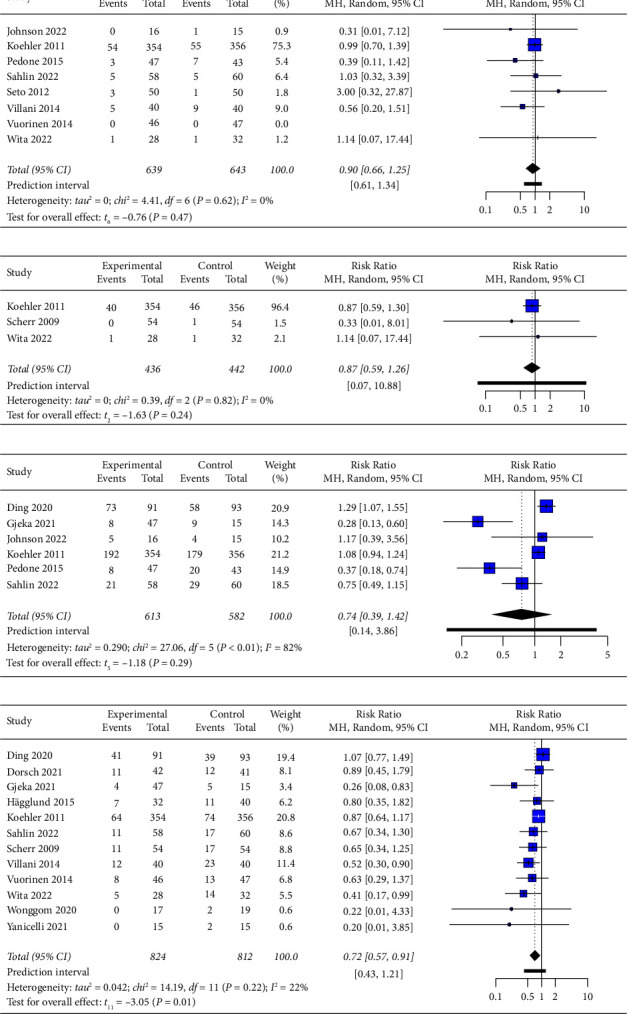
Forest plot of the effect of mobile health application-based interventions on (a) all-cause mortality, (b) cardiovascular mortality, (c) all-cause hospitalization, and (d) heart failure-related hospitalization.

Eight studies provided data regarding all-cause mortality, and the pooled results showed that mobile health application-based interventions had no statistically significant effect on all-cause mortality (RR = 0.90, 95% CI 0.66 to 1.25, *p*=0.47, moderate-certainty evidence; Figure 3(a) and Supplementary Material Table S3), with no evidence of heterogeneity (*I*^2^ = 0%, *p*=0.62).

#### 3.6.2. Cardiovascular Mortality

Three studies reported cardiovascular mortality as an outcome, and the pooled results showed that mobile health application-based interventions had no statistically significant effect on cardiovascular mortality (RR = 0.87, 95% CI 0.59 to 1.26, *p*=0.24, moderate-certainty evidence; Figure 3(b) and Supplementary Material Table S3), with no evidence of heterogeneity (*I*^2^ = 0%, *p*=0.82).

#### 3.6.3. All-Cause Hospitalization

Seven studies assessed the effectiveness of mobile health application-based interventions for all-cause hospitalization, but only six studies were included in the meta-analysis. The excluded studies [39] reported the means and standard deviations instead of the numbers and percentages. The pooled results indicated that mobile health application-based interventions had no statistically significant effect on all-cause hospitalization (RR = 0.74, 95% CI 0.39 to 1.42, *p*=0.29, low-certainty evidence; Figure 3(c) and Supplementary Material Table S3), with statistically significant heterogeneity (*I*^2^ = 82%, *p* < 0.01). Seto et al. [39] reported no group differences in all-cause hospitalization.

#### 3.6.4. Heart Failure-Related Hospitalization

Thirteen studies reported heart failure-related hospitalization. One study [17] was excluded from the meta-analysis because it reported the median hospitalization duration rather than the number and percentage of patients. The pooled results showed that mobile health application-based interventions significantly reduced heart failure-related hospitalization (RR = 0.72, 95% CI 0.57 to 0.91, *p*=0.01, low-certainty evidence; Figure 3(d) and Supplementary Material Table S3), with unimportant heterogeneity (*I*^2^ = 22%, *p*=0.22). This effect size corresponds to an NNT of 14, indicating that one hospitalization from heart failure could be expectantly averted for every 14 people treated. The sensitivity analysis showed that the pooled result was not altered after removing the included studies one by one, indicating the robustness of the result.

#### 3.6.5. Self-Care

**Figure 4:**
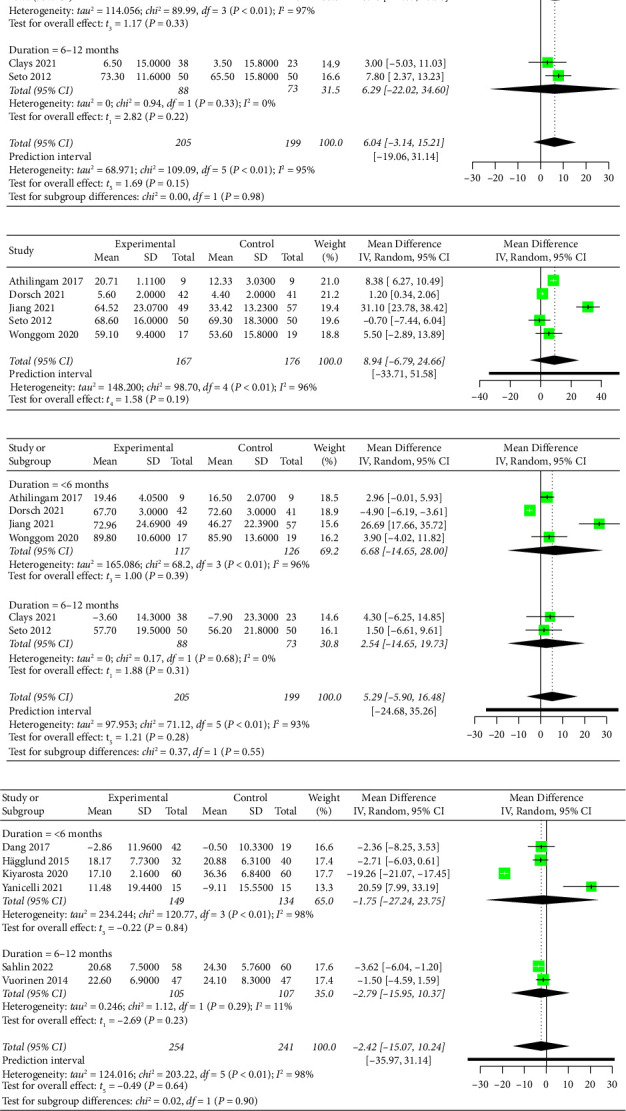
Forest plot of the effect of mobile health application-based interventions on (a) self-care maintenance, (b) self-care management, (c) self-care confidence, and (d) overall self-care.

Twelve studies assessed the effectiveness of mobile health application-based interventions for self-care. Six studies using the SCHFI reported scores for each subscale (i.e., self-care maintenance, self-care management, and self-care confidence) instead of the total score. Six studies used EHFScB to evaluate self-care. Higher SCHFI scores represented better self-care, while higher EHFScB scores indicated worse self-care. Additionally, the score of SCHFI was commonly presented in the subdimension rather than the total score. Thus, the effectiveness of self-care was separately combined based on different tools. The pooled results demonstrated that mobile health application-based intervention had no statistically significant effect on self-care maintenance (MD = 6.04, 95% CI −3.14 to 15.21, *p*=0.15, low-certainty evidence; Figure 4(a) and Supplementary Material Table S3), self-care management (MD = 8.94, 95% CI −6.79 to 24.66, *p*=0.19, very low-certainty evidence; Figure 4(b) and Supplementary Material Table S3), or self-care confidence (MD = 5.29, 95% CI −5.90 to 16.48, *p*=0.28, low-certainty evidence; Figure 4(c) and Supplementary Material Table S3) when measured using the SCHFI. Furthermore, the pooled results showed no statistically significant effects on overall self-care when measured using the EHFScB (MD = −2.42, 95% CI −15.07 to 10.24, *p*=0.64, low-certainty evidence; Figure 4(d) and Supplementary Material Table S3).

#### 3.6.6. Quality of Life

**Figure 5:**
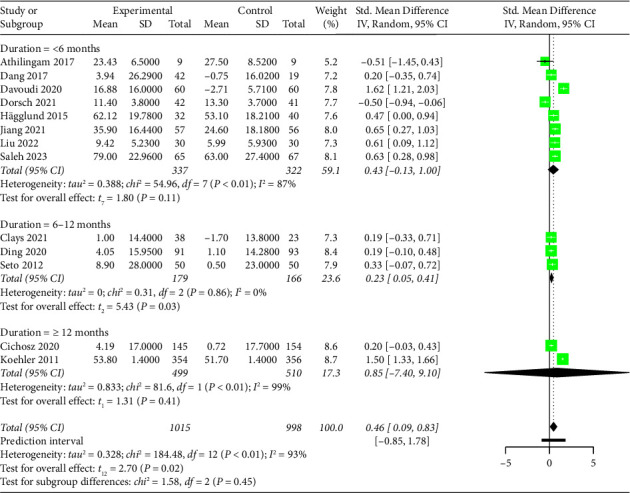
Forest plot of the effect of mobile health application-based interventions on quality of life.

Fourteen studies evaluated the effectiveness of mobile health application-based interventions on quality of life. Twelve studies used one tool to measure quality of life, with six using the MLHFQ, three using the KCCQ, two using the SF-36, and one using the EuroQoL Five-Dimension. Two studies used two tools (the SF-36 and MLHFQ [45] and the SF-36 and KCCQ [46]) to evaluate quality of life. For these two studies, the MLHFQ or KCCQ score were used to perform the meta-analysis instead of the SF-36 because the SF-36 score was presented subdimensionally rather than as the total score. Among the 14 studies measuring quality of life, one study [51] was excluded from the meta-analysis due to incomplete data. Because lower scores on the MLHFQ indicate better quality of life, while higher scores on other tools indicate better quality of life, the changes in quality of life using MLHFQ were recalculated in the present study using the baseline score minus the final score based on the Cochrane Handbook [28] and a previous meta-analysis [54] to obtain a consistent explanation of the scale scores. The pooled results showed that mobile health application-based interventions had a small but statistically significant effect on improving quality of life (SMD = 0.46, 95% CI 0.09 to 0.83, *p*=0.02, low-certainty evidence; Figure 5 and Supplementary Material Table S3), with substantial heterogeneity (*I*^2^ = 93%, *p* < 0.01). The sensitivity analysis demonstrated that the pooled result was opposite after removing the study [18], probably because the baseline quality of life in this study was lower than that in other studies.

#### 3.6.7. Subgroup Analysis

A series of subgroup analyses were conducted based on the duration of intervention (<6 months, 6–12 months, or ≥12 months), mobile device (smartphone, mobile phone, or others), frequency of monitoring (daily or not daily), and delivery personnel (multidisciplinary team, not multidisciplinary team, or not specified). For the self-care subscales from the SCHFI (self-care maintenance and self-care confidence) and overall self-care from the EHFScB, the effect sizes among subgroups were similar, and no significant subgroup differences (*Q* = 0.00–2.83, *p*=0.24–0.98) were found except for different mobile devices for overall self-care (*Q* = 8.39, *p*=0.02) (Figure 4 and Supplementary Material Table S4). No subgroup analysis was performed for self-care management (subscale of the SCHFI) due to the small number of studies included [55].

For quality of life, the results showed that mobile health application-based interventions were effective for improving quality of life when the duration of the intervention was between 6 and 12 months (SMD = 0.23, 95% CI 0.05 to 0.41, *p*=0.03); however, no statistically significant effect occurred when the intervention duration was less than 6 months (SMD = 0.43, 95% CI −0.13 to 1.00, *p*=0.11) or more than 12 months (SMD = 0.85, 95% CI −7.40 to 9.10, *p*=0.41) (Figure 5). Nevertheless, the subgroup effect was not statistically significant (*Q* = 1.58, *p*=0.45). According to the subgroup analysis of the frequency of monitoring, mobile health application-based interventions had a significant effect on quality of life when physiological data were monitored daily (SMD = 0.47, 95% CI 0.02 to 0.92, *p*=0.04); however, no statistically significant effect was found when physiological data were not monitored daily (SMD = 0.40, 95% CI −2.26 to 3.05, *p*=0.31) (Supplementary Material Table S4). No subgroup differences were detected (*Q* = 0.06, *p*=0.80).

#### 3.6.8. Publication Bias

Egger's regression intercept indicated that publication bias in quality of life was not statistically significant (*p*=0.054). However, publication bias was found for heart failure-related hospitalization according to Egger's regression intercept (*p*=0.004). Funnel plots of heart failure-related hospitalization and quality of life are presented in Supplementary Material Figures S1 and S2, respectively. Funnel plots for all-cause mortality, cardiovascular mortality, all-cause hospitalization, and self-care were not generated due to the small number of studies [28].

## 4. Discussion

This systematic review and meta-analysis summarized evidence from 24 studies involving 2886 participants to identify the effects of mobile health application-based interventions among people with heart failure. The pooled results showed that mobile health application-based interventions had beneficial effects on reducing heart failure-related hospitalization and improving quality of life but had no statistically significant effect on all-cause mortality, cardiovascular mortality, all-cause hospitalization, or self-care.

The results indicated that application-based interventions could not reduce mortality or all-cause hospitalization. These findings are consistent with previous reviews [26], which revealed no statistically significant positive effect of mobile telemonitoring applications on mortality in people with heart failure. Similarly, a review focused on mobile phone-based interventions for managing heart failure revealed that such interventions were not effective at reducing all-cause mortality or all-cause hospitalization [22]. One possible reason could be that almost all interventions included in this review monitored indices specific to heart failure (e.g., weight and heart failure symptoms), which may limit the possibility of identifying exacerbations related to other chronic conditions (e.g., diabetes and chronic obstructive pulmonary disease) and other cardiovascular diseases (e.g., ventricular arrhythmias) [21]. Another possible explanation is that more than half of the participants were older adults who had a high risk of comorbidity [56], prompting hospitalization because of another disease instead of heart failure.

The findings suggested that the application-based interventions may have benefits for reducing heart failure-related hospitalization, which was in line with the findings of a previous study [49]. Most of the included studies in this review had the feature of self-monitoring and feedback. Self-monitoring of physiological data (e.g., weight, blood pressure, and heart rate) and heart failure symptoms allows healthcare providers to track patients' health information and provide timely suggestions and knowledge about disease [49, 50], which helps patients take early action to delay the worsening of heart failure, resulting in decreased failure-related hospitalization. However, these findings should be interpreted with caution. First, the certainty of evidence was low, and the included studies had varied risks of bias. Second, publication bias was detected for the outcome of heart failure-related hospitalization, which may overestimate the effectiveness of application-based interventions.

The results of the meta-analysis indicated that the effects of application-based interventions on self-care were not observed, whether measured with the SCHFI or EHFScB. These findings are supported by those of Wonggom et al. [19], who reported that the use of application-based interventions had no statistically significant effect on self-care maintenance or self-care confidence. However, these findings were inconsistent with those of a previous review by Zhang et al. [57]. A possible explanation for this difference is that nearly half of the included studies in this review did not provide additional health education resources, which may have limited the improvement of heart failure-related knowledge and skills and was less likely to cause statistically significant impacts on self-care. Nevertheless, almost all the included studies in Zhang et al.'s [57] review provided self-management guidance for chronic heart failure, resulting in improved self-care. Hence, our results suggest that health education can be embedded in applications in addition to tele-monitoring features and offers much more visual and vivid education, for example, videos, questions and answer games [50]. In addition, the studies included in this review rarely used empowerment strategies or behavior change theories, which may have resulted in a nonsignificant effect on self-care behavior in the present review, as incorporating empowerment strategies and behavior change theories into interventions is more effective than not incorporating such strategies [58]. Therefore, future studies should be developed based on empowerment strategies and behavior change theories.

Despite the apparent heterogeneity, this review revealed a statistically significant effect of mobile health application-based interventions on improving quality of life. Similarly, Zhang et al. [57] reported that electronic health interventions, such as application-based interventions, effectively increase quality of life in people with chronic heart failure. However, our findings are inconsistent with those of Son et al. [22] and Kitsiou et al. [21]. These differences may be because previous reviews included various mobile health technologies, such as voice call interventions and short message services, while the current review limited the intervention to mobile applications. Moreover, the most frequent intervention was voice calls in Son et al.'s study, which lacked an interface for telemonitoring interactions [26]. Self-monitoring, reminders, and feedback were the common features of our included studies; these features make it easy for patients to receive immediate support from healthcare professionals, eventually leading to improved quality of life. Subgroup analysis revealed that the effect on quality of life was observed when patients received mobile health application-based interventions for 6–12 months or when self-monitoring was conducted daily. However, the subgroup effect was not statistically significant, which might be due to uneven distributions of studies and participants across subgroups [59]. This result should be interpreted with caution because of the small number of long-term studies (two to three trials), the heterogeneity of the interventions, and the low certainty of the evidence. Hence, further rigorous trials are warranted to draw highly robust conclusions.

Notably, when delivering mobile health application-based interventions for people with heart failure, the acceptance and simplicity of use of the applications need to be considered [60], especially for elderly patients and people with limited digital literacy [61]. The characteristics of applications may reduce patient engagement and increase the attrition rate, which in turn affects intervention effectiveness [61].

### 4.1. Limitations

This systematic review has several limitations. First, the methodological qualities of the included studies were not optimal, and all studies showed performance bias due to the nature of the interventions. Second, there was substantial heterogeneity between studies due to differences in sample sizes, intervention characteristics (e.g., application features, duration and frequency of monitoring), participant characteristics, and assessment tools. Therefore, the results of the current study must be interpreted with caution. Third, the included studies were mostly conducted in developed countries, which may limit the generalizability of our results. Fourth, only English-language studies were included, which may result in possible publication bias. Finally, cautions should be taken when using the evidence, as the overall GRADE evidence was very low to moderate.

### 4.2. Implications

This systematic review provides a quantitative synthesis of the effects of mobile health application-based interventions on mortality, hospitalization, self-care, and quality of life. The findings suggest that the use of mobile health application-based interventions can be considered a useful strategy for managing heart failure. Given the prevalence of smartphone ownership [62], mobile applications are a viable option for patients to obtain medical suggestions and support. Existing studies that involve application-based interventions differ in terms of delivery devices, personnel, key features of the application, durations, and outcome measures [18, 20]. Therefore, the optimal component of mobile health application-based interventions should be examined in the future based on patient characteristics and multicultural contexts. Only eight out of 24 studies used empowerment strategies and behavior change theories. Hence, additional theory-guided trials are warranted to determine the impact of mobile health application-based interventions on self-care. Due to the complexity of heart failure, a multidisciplinary team is recommended for delivery application-based interventions.

## 5. Conclusions

This systematic review and meta-analysis showed that mobile health application-based interventions are effective at reducing heart failure-related hospitalization and improving quality of life. Evidence was not conclusive for mortality, self-care, and all-cause hospitalization. The effects on quality of life may vary among the different durations of the interventions and frequency of monitoring. Hence, well-designed randomized controlled trials are needed to explore the optimal duration of intervention and strengthen the current evidence.

## Data Availability

The data used to support the findings of this study are available from the corresponding authors upon reasonable request.

## Supplementary Materials

Supplementary Materials Table S1: Search strategy. Table S2: Characteristics of the included studies. Table S3: GRADE certainty grading evaluation. Table S4: Subgroup analyses of mobile health application-based interventions on self-care and quality of life. Figure S1: Funnel plots of heart failure-related hospitalization. Figure S2: Funnel plots of quality of life.
