# Supplementary material for: Mobile Application-Based Interventions for People with Heart Failure: A Systematic Review and Meta-Analysis
Source: J Nurs Manag. 2024 Jul 29;2024:6859795. doi: 10.1155/2024/6859795 (PMC11918826; doi:10.1155/2024/6859795)
Supplement: Supplementary Materials — Table S1: Search strategy. Table S2: Characteristics of the included studies. Table S3: GRADE certainty grading evaluation. Table S4: Subgroup analyses of mobile health application-based interventions on self-care and quality of life. Figure S1: Funnel plots of heart failure-related hospitalization. Figure S2: Funnel plots of quality of life. [file 6859795.f1.docx]

**Supplemental materials**

Table S1 Search strategy

| Databases | Search No. | Index terms and Keywords |
| --- | --- | --- |
| MEDLINE | #1 | Heart failure[MeSH Terms] |
|  | #2 | (((((Heart failure[Title/Abstract]) OR (cardiac failure[Title/Abstract])) OR (congestive heart failure[Title/Abstract])) OR (chronic heart failure[Title/Abstract])) OR (myocardial failure[Title/Abstract])) OR (Heart Decompensation[Title/Abstract]) |
|  | #3 | #1 and #2 |
|  | #4 | Mobile Health[MeSH Terms] |
|  | #5 | Cell Phone[MeSH Terms] |
|  | #6 | Mobile Applications[MeSH Terms] |
|  | #7 | (((((((((Mobile Health[Title/Abstract]) OR (smart phone[Title/Abstract])) OR (Cell Phone[Title/Abstract])) OR (mobile phone[Title/Abstract])) OR (Mobile Applications[Title/Abstract])) OR (mobile app[Title/Abstract])) OR (mobile apps[Title/Abstract])) OR (electronic application[Title/Abstract])) OR (mobile application[Title/Abstract])) OR (apps[Title/Abstract]) |
|  | #8 | #4 and #5 and #6 and #7 |
|  | #9 | #3 and #8 |
|  | #10 | #9 and (English[Language]) |
| CINAHL **Plus with Full Text** | S1 | SU  Heart failure |
|  | S2 | SU cardiac failure |
|  | S3 | SU congestive heart failure |
|  | S4 | SU chronic heart failure |
|  | S5 | SU myocardial failure |
|  | S6 | SU Heart Decompensation |
|  | S7 | S1 OR S2 OR S3 OR S4 OR S5 OR S6 |
|  | S8 | SU Mobile Health |
|  | S9 | SU celluar phone |
|  | S10 | SU smart phone |
|  | S11 | SU Cell Phone |
|  | S12 | SU mobile phone |
|  | S13 | SU Mobile Applications |
|  | S14 | SU mobile app |
|  | S15 | SU mobile apps |
|  | S16 | SU electronic application |
|  | S17 | SU mobile application |
|  | S18 | S8 OR S9 OR S10 OR S11 OR S12 OR S13 OR S14 OR S15 OR S16 OR S17 |
|  | S19 | S7 AND S18 |
|  | S20 | S19 and LA English |
| PsycINFO | S1 | SU Heart failure |
|  | S2 | SU cardiac failure |
|  | S3 | SU congestive heart failure |
|  | S4 | SU chronic heart failure |
|  | S5 | SU myocardial failure |
|  | S6 | SU Heart Decompensation |
|  | S7 | S1 OR S2 OR S3 OR S4 OR S5 OR S6 |
|  | S8 | SU Mobile Health |
|  | S9 | SU celluar phone |
|  | S10 | SU smart phone |
|  | S11 | SU Cell Phone |
|  | S12 | SU mobile phone |
|  | S13 | SU Mobile Applications |
|  | S14 | SU mobile app |
|  | S15 | SU mobile apps |
|  | S16 | SU electronic application |
|  | S17 | SU mobile application |
|  | S18 | S8 OR S9 OR S10 OR S11 OR S12 OR S13 OR S14 OR S15 OR S16 OR S17 |
|  | S19 | S7 AND S18 |
|  | S20 | limit S19 to English language |
| WOS | #1 | **TS=(Heart failure)** |
|  | #2 | **TS=(cardiac failure)** |
|  | #3 | **TS=(congestive heart failure)** |
|  | #4 | **TS=(chronic heart failure)** |
|  | #5 | **TS=(myocardial failure)** |
|  | #6 | **TS=(Heart Decompensation)** |
|  | #7 | #1 OR #2 OR #3 OR #4 OR #5 OR #6 |
|  | #8 | **TS=(Mobile Health)** |
|  | #9 | TS=(celluar phone) |
|  | #10 | **TS=(smart phone)** |
|  | #11 | **TS=(Cell Phone)** |
|  | #12 | **TS=(mobile phone)** |
|  | #13 | **TS=(Mobile Applications)** |
|  | #14 | **TS=(mobile app)** |
|  | #15 | **TS=(mobile apps)** |
|  | #16 | **TS=(electronic application)** |
|  | #17 | **TS=(mobile application)** |
|  | #18 | **TS=(apps)** |
|  | #19 | **#8 OR #9 OR #10 OR #11 OR #12 OR #13 OR #14 OR #15 OR #16 OR #17** **OR #18** |
|  | #20 | **#7 AND #19** |
|  | #21 | limit #20 to English language |
| EMBASE | 1 | exp heart failure/ |
|  | 2 | Heart failure.ab,ti. |
|  | 3 | cardiac failure.ab,ti. |
|  | 4 | congestive heart failure.ab,ti. |
|  | 5 | chronic heart failure.ab,ti. |
|  | 6 | myocardial failure.ab,ti. |
|  | 7 | Heart Decompensation.ab,ti. |
|  | 8 | 1 or 2 or 3 or 4 or 5 or 6 or 7 |
|  | 9 | exp Cell Phone/ |
|  | 10 | exp Mobile Applications/ |
|  | 11 | Mobile Health.ab,ti. |
|  | 12 | smart phone.ab,ti. |
|  | 13 | Cell Phone.ab,ti. |
|  | 14 | mobile phone.ab,ti. |
|  | 15 | Mobile Applications.ab,ti. |
|  | 16 | mobile app.ab,ti. |
|  | 17 | mobile apps.ab,ti. |
|  | 18 | electronic application.ab,ti. |
|  | 19 | mobile application.ab,ti. |
|  | 20 | apps.ab,ti. |
|  | 21 | 9 or 10 or 11 or 12 or 13 or 14 or 15 or 16 or 17 or 18 or 19 or 20 |
|  | 22 | 8 and 21 |
|  | 23 | limit 22 to English language |
| CENTRAL (Ovid) | 1 | exp heart failure/ |
|  | 2 | Heart failure.ti,kw,ab. |
|  | 3 | cardiac failure. ti,kw,ab. |
|  | 4 | congestive heart failure. ti,kw,ab. |
|  | 5 | chronic heart failure. ti,kw,ab. |
|  | 6 | myocardial failure. ti,kw,ab. |
|  | 7 | Heart Decompensation. ti,kw,ab. |
|  | 8 | 1 or 2 or 3 or 4 or 5 or 6 or 7 |
|  | 9 | exp Mobile Health/ |
|  | 10 | exp Cell Phone/ |
|  | 11 | exp Mobile Applications/ |
|  | 12 | Mobile Health. ti,kw,ab. |
|  | 13 | smart phone. ti,kw,ab. |
|  | 14 | Cell Phone. ti,kw,ab. |
|  | 15 | mobile phone. ti,kw,ab. |
|  | 16 | Mobile Applications. ti,kw,ab. |
|  | 17 | mobile app. ti,kw,ab. |
|  | 18 | mobile apps. ti,kw,ab. |
|  | 19 | electronic application. ti,kw,ab. |
|  | 20 | mobile application. ti,kw,ab. |
|  | 21 | apps. ti,kw,ab. |
|  | 22 | 9 or 10 or 11 or 12 or 13 or 14 or 15 or 16 or 17 or 18 or 19 or 20 or 21 |
|  | 23 | 8 and 22 |
|  | 24 | limit 23 to English language |

Table S2 Characteristics of the included studies.

| Author  (year) Country | Study Design and sample size (Intervention vs. control) | Participants' characteristics (Intervention vs. control) | Intervention group | Control group | Duration/Follow-up (Attrition rate) | Outcomes (instrument) | Grant |
| --- | --- | --- | --- | --- | --- | --- | --- |
| Scherr et al.  (2009)  Austria | 2-arm RCT  Total: 108  54 vs. 54 | Age: 65 (62-72) vs. 67 (61-72)  Female: 26% vs. 28%  NYHA Ⅲ-Ⅳ: 87% vs. 87%  LVEF (%): 25 vs. 29 | **Key features:** reminders and notifications**,** self-monitoring (manual) and assessment, feedback and alerts (immediate)  **Frequency:** daily monitoring for blood pressure, heart rate, and weight  **Mobile** **device:** mobile phone  **Delivery personnel:** physician | Standard care (pharmacological treatment) | 6 months/6 months (27.8%) | Cardiovascular mortality  HF-related hospitalization | Yes |
| Koehler et al.  (2011)  Germany | 2-arm RCT  Total: 710  354 vs.356 | Age: 66.9±10.8 vs. 66.9±10.5  Female: 19.5% vs. 18.0%  NYHA Ⅲ-Ⅳ: 50.3% vs. 49.4%  LVEF (%): 26.9±5.7 vs. 27.0±5.9 | **Key features:** self-monitoring (automatic) and assessment, tele-coaching, feedback and alerts (immediate)  **Frequency:** daily monitoring for blood pressure, weight, and ECG  **Type of mobile device:** PDA  **Delivery personnel:** physician | Usual care | 24 months/24 months (16.5%) | All-cause mortality  Cardiovascular mortality  All-cause hospitalization  HF-related hospitalization  QoL (SF-36) | Yes |
| Seto et al.  (2012)  Canada | 2-arm RCT  Total: 100  50 vs. 50 | Age: 55.1±13.7 vs. 52.3 ±13.7  Female: 18% vs. 24%  NYHA Ⅲ-Ⅳ: 46% vs. 46%  LVEF (%): 27.1±7.8 vs. 27.0 ±9.9 | **Key features:** reminders and notifications, self-monitoring (automatic) and assessment, feedback and alerts (immediate), tele-coaching  **Frequency:** daily monitoring for blood pressure, weight, and symptoms, weekly for ECG  **Mobile device:** mobile phone  **Delivery personnel:** Multidisciplinary team (cardiologist and nurse practitioner) | Standard care | 6 months/6 months (16%) | All-cause mortality  All-cause hospitalization  Self-care (SCHFI)  QoL (MLHFQ) | Yes |
| Villani et al.  (2014)  Italy | 2-arm RCT  Total: 80  40 vs. 40 | Age: 71± 4 vs. 73± 5  Female: 10% vs. 45%  NYHA (score): 3.08±0.57 vs. 2.90 ± 0.69  LVEF (%): < 40% (all of participants) | **Key features:** reminders and notifications, self-monitoring and assessment (automatic), feedback and alerts (delayed), tele-coaching  **Frequency:** daily monitoring for blood pressure and weight, weekly for the ECG  **Mobile device:** PDA  **Delivery personnel:** cardiologist | Usual Care | 12 months/12 months (NR) | All-cause mortality  HF-related hospitalization | Yes |
| Vuorinen et al.  (2014)  Finland | 2-arm RCT  Total: 94  47 vs. 47 | Age: 58.3±11.6 vs. 57.9 ±11.9  Female: 60% vs. 64%  NYHA Ⅲ-Ⅳ: 98% vs. 96%  LVEF (%): 27.3±4.9 vs. 28.6±5.0 | **Key features:** self-monitoring and assessment (manual), feedback and alerts (immediate)  **Frequency:** monitoring for blood pressure, pulse, weight together with the assessment of symptoms once a week  **Mobile device:** mobile phone  **Delivery personnel:** nurse | Usual Care | 6 months/6 months (1.1%) | All-cause mortality  HF-related hospitalization  Self-care (EHFScB) | Yes |
| Hägglund et al.  (2015)  Sweden | 2-arm RCT  Total: 72  32 vs. 40 | Age: 75±8 vs. 76±7  Female: 34% vs. 30%  NYHA Ⅲ-Ⅳ: 62% vs. 82%  LVEF (%): NR | **Key features:** self-monitoring (automatic) and assessment, health education  **Frequency:** daily monitoring for weight and symptoms  **Mobile device:** tablet  **Delivery personnel:** Multidisciplinary team (nurses and doctors at the HF center) | Standard care | 3 months/3 months (13.9%) | HF-related hospitalization  Self-care (EHFScB)  QoL (KCCQ) | Yes |
| Pedone et al.  (2015)  Italy | 2-arm RCT  Total: 90  47 vs.43 | Age: 79.9±6.8 vs. 79.7±7.8  Female: 53.2% vs. 69.8%  NYHA Ⅲ-Ⅳ: 68.0% vs. 67.4%  LVEF (%):44.4±12.7 vs. 48.2±13.5 | **Key features:** self-monitoring (automatic) and assessment, feedback and alerts (immediate), tele-coaching  **Frequency:** weight once a day; blood pressure and heart rate twice a day, and peripheral oxygen saturation three times a day  **Mobile device:** smartphone  **Delivery personnel:** geriatrician | Standard Care | 6 months/6 months (6.25%) | All-cause mortality  All-cause hospitalization | No |
| Athilingam et al.  (2017)  US | 2-arm pilot RCT  Total: 18  9 vs. 9 | Age (total): 53.06 ±4.02  Female (total): 55.6%  NYHA Ⅲ-Ⅳ(total): 33.3%  LVEF (%) (total): 28 | **Key features:** reminders and notifications, self-monitoring (automatic) and assessment, health education, feedback and alerts (immediate), physiological exercises, tele-coaching  **Frequency:** daily monitoring for weight, blood pressure, symptoms, and vital sign monitoring (heart rate and accelerometer data)  **Mobile device:** smartphone  **Delivery personnel:** home health nurses | Waitlist control | 4 weeks/30 day (28%) | Self-care (SCHFI)  QoL (KCCQ) | Yes |
| Dang et al.  (2017)  US | 2-arm RCT  Total: 61  42 vs. 19 | Age: 53.0 ± 9.4 vs. 60.3 ± 9.0  Female: 33.3% vs. 42.1%  NYHA Ⅲ-Ⅳ:16.7% vs. 10.5%  LVEF≤40%: 69.0% of the participants vs. 57.9% participants | **Key features:** reminders and notifications, self-monitoring (manual) and assessment, feedback and alerts (immediate)  **Frequency:** daily monitoring for weight and symptoms  **Mobile device:** mobile phone  **Delivery personnel:** study coordinator | Usual Care | 3 months/3 months (14.8%) | Self-care (EHFScB)  QoL (MLHFQ, SF-36) | Yes |
| Cichosz et al.  (2020)  Denmark | 2-arm RCT  Total: 299  145 vs. 154 | Age: 70 (59.5; 77) vs. 69 (61; 76)  Female: 17% vs. 21%  NYHA (score, median [25; 75 percentile]): 2 [2; 3] vs. 2 [2; 3]  LVEF (%): NR | **Key features:** reminders and notifications, self-monitoring (automatic) and assessment, feedback and alerts (delayed)  **Frequency:** daily monitoring for weight and blood pressure during the first 2 weeks and one to two times weekly after the first 2 weeks  **Mobile device:** tablet  **Delivery personnel:** trained nurses | Usual care | 12 months/12 months (35.5%) | QoL (KCCQ, SF-36) | Yes |
| Davoudi et al.  (2020)  Iran | 2-arm RCT  Total: 120  60 vs. 60 | Age: 50.07±11.77 vs. 52.78±12.2  Female: 38% vs. 46%  NYHA: Ⅱ-Ⅲ (all of participants)  LVEF (%): NR | **Key features:** reminders and notifications, self-monitoring (manual) and assessment, health education, feedback and alerts, interaction  **Frequency:** daily monitoring for vital signs, symptoms, and weight  **Mobile device:** smartphone  **Delivery personnel:** nurses | Usual care | 3 months/3months (7.5%) | QoL (MLHFQ) | Yes |
| Ding et al.  (2020)  Australia | 2-arm RCT  Total: 184  91 vs. 93 | Age: 69.5±12.3 vs. 70.8 ±12.4  Female: 27% vs. 19%  NYHA (score): 2.0±0.5 vs. 2.2±0.6  LVEF (%): 29.1±7.1 vs. 27.4±15.9 | **Key features:** reminders and notifications, self-monitoring (automatic) and assessment, feedback and alerts (immediate)  **Frequency:** daily monitoring for weight  **Mobile device:** tablet  **Delivery personnel:** project nurses | Usual care | 6 months/6 months (19.6%) | All-cause hospitalization  HF-related hospitalization  QoL (EQ-5D) | Yes |
| Kiyarosta et al.  (2020)  Iran | 2-arm RCT  Total: 120  60 vs. 60 | Age: 55.95±14.41 vs. 60.71±12.62  Female: 48.3% vs. 41.7%  NYHA: Ⅱ-Ⅲ (all of participants)  LVEF (%): 25±8.33 vs. 22.75±8.45 | **Key features:** reminders and notifications, self-monitoring (manual) and assessment, health education, interaction  **Frequency:** daily monitoring for weight, vital signs, and symptoms  **Mobile device:** smartphone  **Delivery personnel:** nurses | Usual care | 3 months/3 months (0%) | Self-care (EHFScB) | NR |
| Wonggom et al.  (2020)  Thailand | 2-arm RCT  Total: 36  17 vs. 19 | Age: 68.7 ±11.6 vs. 66.6 ±11.3  Female: 17.6% vs. 21.1%  NYHA Ⅲ-Ⅳ: 0% vs. 15.8%  LVEF < 40%: 29.4% of the participants vs. 36.8% participants | **Key features:** health education  **Frequency:** using the app as often, or as little as participants would like to  **Mobile device:** tablet  **Delivery personnel:** Not specified | Usual care | 90 days/90 days  (2.8%) | Self-care (SCHFI)  HF-related hospitalization | Yes |
| Clays et al.  (2021)  Belgium | 2-arm RCT  Total: 56  34 vs. 22 | Age: 61.8±11.0 vs. 65.2 ±9.6  Female: 23.5% vs. 22.7%  NYHA Ⅲ-Ⅳ: 16.1% vs. 9.1%  LVEF (%):32.7 ±5.9 vs. 31.3 ±6.9 | **Key features:** reminders and notifications, self-monitoring (automatic) and assessment, goal-setting, health education, psychological support (cognitive behavioral therapy and mindfulness exercises), feedback and alerts (immediate), tele-coaching  **Frequency:** monitoring for blood pressure, heart rate, and respiratory rate twice a week, monitoring for weight once a day or twice a week (configurable by physician)  **Mobile device:** smartphone  **Delivery personnel:** Multidisciplinary team (cardiologist, general practitioner and CHF nurse) | Usual care | 6 months/6 months (8.2%) | Self-care (SCHFI)  QoL (MLHFQ) | Yes |
| Dorsch et al.  (2021)  US | 2-arm RCT  Total: 83  42 vs. 41 | Age: 60.2 ±9 vs. 62 ±9  Female: 33% vs. 37%  NYHA Ⅲ-Ⅳ: 74% vs. 88%  LVEF (%):37.2 ±20 vs. 38.8 ±19 | **Key features:** reminders and notifications, self-monitoring and assessment (manual), health education, feedback and alerts (immediate)  **Frequency:** daily monitoring for weight  **Mobile device:** smartphone  **Delivery personnel:** Not specified | Usual care | 12 weeks/12 weeks (2.4%) | HF-related hospitalization  Self-care (SCHFI)  QoL (MLHFQ) | Yes |
| Gjeka et al.  (2021)  US | 2-arm RCT  Total: 62  47 vs. 15 | Age: 68.1 vs. 70.0  Female: 51.1% vs. 33.3%  NYHA Ⅲ-Ⅳ: 100%  LVEF (%): NR | **Key features:** reminders and notifications, self-monitoring (automatic and manual) and assessment, health education, feedback and alerts (immediate)  **Frequency:** daily monitoring for blood pressure, heart rate, blood-oxygen saturation, weight, BMI, and symptoms  **Mobile device:** smartphones  **Delivery personnel:** clinicians | Usual care | 45 days/45 days (NR) | All-cause hospitalization  HF-related hospitalization | No |
| Jiang et al.  (2021)  Singapore | 3-arm RCT  Total: 162  IG A:49  IG B:57  CG:56 | Age: 66.82 ±11.81 vs. 68.82 ±13.14  Female: 29.8% vs. 28.5%  NYHA Ⅲ-Ⅳ: 71.9% vs. 69.6%  LVEF (%): NR | **Key features:** reminders and notifications, self-monitoring and assessment, health education, feedback and alerts, interaction, motivational interviewing  **Frequency:** daily monitoring for weight, blood pressure and symptom  **Mobile device:** smartphone  **Delivery personnel:** research nurse | Usual care | 6 weeks/6 months (23.9%) | HF-related hospitalization  Self-care (SCHFI)  QoL (MLHFQ) | Yes |
| Yanicelli et al.  (2021)  Argentina | 2-arm RCT  Total: 30  15 vs. 15 | Age (total): 52 years  Female: 34% vs. 7%  NYHA Ⅲ-Ⅳ: 32% vs. 26%  LVEF (%): 35.93±12.13 vs. 29.8±7.23 | **Key features:** self-monitoring and assessment (manual), health education, feedback and alerts (delayed)  **Frequency:** daily monitoring for weight, blood pressure, heart rate and symptoms  **Mobile device:** smartphone  **Delivery personnel:** Multidisciplinary team (four cardiologists and a nurse) | Usual care | 3 months/3 months (25%) | HF-related hospitalization  Self-care (EHFScB) | No |
| Johnson et al.  (2022)  US | 2-arm pilot RCT  Total: 31  16 vs. 15 | Age: 60.1±12.9 vs. 60.7 ±15.0  Female: 37.5% vs. 46.7%  NYHA Ⅲ-Ⅳ: NR  LVEF < 40%: 62.5% of the participants vs. 46.7% of participants | **Key features:** reminders and notifications, self-monitoring and assessment, health education, feedback and alerts  **Frequency:** daily monitoring for symptoms  **Mobile device:** smartphone  **Delivery personnel:** nurses | Usual care | 90 days/90 days (9.7%) | All-cause mortality  All-cause hospitalization  QoL (KCCQ) | Yes |
| Liu et al. (2022)  China | 2-arm RCT  Total: 60  30 vs. 30 | Age: 53.27± 7.1 vs. 55.27± 6.01  Female: 43.3%vs. 40.0%  NYHA: Ⅱ-Ⅲ (all of participants)  LVEF (%): 36.02± 5.12 vs. 35.41± 6.17 | **Key features:** reminders and notifications, self-monitoring (automatic) and assessment, feedback and alerts (immediate), health education, tele-coaching  **Frequency:** monitoring for heart rate, exercise speed, exercise time, and other parameters in real time, weekly exercise course  **Mobile device:** smartphone  **Delivery personnel:** Multidisciplinary team (cardiovascular physician and nurse) | Usual care | 12 weeks/12 weeks (NR) | QoL (MLHFQ) | Yes |
| Sahlin et al.  (2022)  Sweden | 2-arm RCT  Total: 118  58 vs. 60 | Age: 80 ± 8 vs. 77 ± 11  Female: 33% vs. 47%  NYHA Ⅱ-Ⅲ: 26% vs. 32%  LVEF < 40%: 46.0% of the participants vs. 45.0% of participants | **Key features:** self-monitoring (automatic) and assessment, health education, feedback and alerts (delayed)  **Frequency:** monitoring for weight, dose of loop diuretics, and a brief education, assessment for symptoms every 5 days  **Mobile device:** tablet  **Delivery personnel:** physician | Standard care | 240 days/240 days (4.8%) | All-cause mortality  All-cause hospitalization  HF-related hospitalization  Self-care (EHFScB) | NR |
| Wita et al.  (2022)  Poland | 2-arm RCT  Total: 60  28 vs. 32 | Age: 65.1±11.7 vs. 66.9 ±9.3  Female: 17.9% vs. 25.0%  NYHA (score, median [25; 75 percentile]): 2 (2–3) vs. 3 (2–3)  LVEF (%): 26.6±7 vs. 26.1±6.7 | **Key features:** self-monitoring (automatic and manual) and assessment, goal-setting, tele-coaching  **Frequency:** daily monitoring for weight, blood pressure and symptoms, weekly for ECG  **Mobile device:** tablet  **Delivery personnel:** physician | Standard care | 24 months/24 months (4.8%) | All-cause mortality  Cardiovascular mortality  HF-related hospitalization | NR |
| Saleh et al.  (2023)  Jordan | 2-arm RCT  Total: 132  65 vs. 67 | Age: 62.2 ± 11.35 vs. 59.4 ± 9.42  Female: 41.5% vs. 37.3%  NYHA Ⅲ-Ⅳ: 46.2% vs. 40.3%  LVEF ≤ 40%: 83.1% of the participants vs. 77.6% of participants | **Key features:** self-monitoring and assessment, goal-setting, interaction, feedback and alerts (delayed)  **Frequency:** daily goal setting for physical activity level, daily step count  **Mobile device:** mobile phone  **Delivery personnel:** cardiologists | Usual care | 8 weeks/8 weeks (13.2%) | QoL (SF-36) | No |

Abbreviations: RCT, randomized controlled trial; NYHA, New York Heart Association; LVEF, left ventricle ejection fraction; NR, not reported; HF, heart failure; PDA, personal digital assistant; QoL, quality of life; SF-36, 36-item Short-form Health Survey; SCHFI, Self-Care of Heart Failure Index; EHFScB, European Heart Failure Self-care Behavior Scale; MLHFQ, Minnesota Living with Heart Failure Questionnaire; KCCQ, Kansas City Cardiomyopathy Questionnaire; EQ-5D, EuroQoL Five-Dimension.

Table S3 GRADE certainty grading evaluation.

| Outcomes | Certainty assessment | | | | | | | No. of participants | Effect (95% CI) | Certainty |
| --- | --- | --- | --- | --- | --- | --- | --- | --- | --- | --- |
|  | **Number of Studies** | Study design | Risk of bias | Inconsistency | Indirectness | Imprecision | Other considerations |  |  |  |
| All-cause mortality | 8 | randomized trial | serious ^a^ | not serious | not serious | not serious | none | 1282 | RR 0.90 (0.66 to 1.25) | ⨁⨁⨁◯  moderate |
| Cardiovascular mortality | 3 | randomized trial | serious ^a^ | not serious | not serious | not serious | none | 878 | RR 0.87 (0.59 to 1.26) | ⨁⨁⨁◯  moderate |
| All-cause hospitalization | 6 | randomized trial | serious ^a^ | serious ^b^ | not serious | not serious | none | 1195 | RR 0.74 (0.39 to 1.42) | ⨁⨁◯◯  low |
| Heart failure-related hospitalization | 12 | randomized trial | serious ^a^ | not serious | not serious | not serious | publication bias strongly suspected | 1636 | RR 0.72 (0.57 to 0.91) | ⨁⨁◯◯  low |
| Self-care maintenance | 6 | randomized trial | serious ^a^ | serious ^b^ | not serious | not serious | none | 404 | MD 6.04 (-3.14 to 15.21) | ⨁⨁◯◯  low |
| Self-care management | 5 | randomized trial | serious ^a^ | serious ^b^ | not serious | serious ^c^ | none | 343 | MD 8.94 (-6.79 to 24.66) | ⨁◯◯◯  very low |
| Self-care confidence | 6 | randomized trial | serious ^a^ | serious ^b^ | not serious | not serious | none | 404 | MD 5.29 (-5.90 to 16.48) | ⨁⨁◯◯  low |
| Overall self-care | 6 | randomized trial | serious ^a^ | serious ^b^ | not serious | not serious | none | 492 | MD -2.42 (-15.07 to 10.24) | ⨁⨁◯◯  low |
| Quality of life | 13 | randomized trial | serious ^a^ | serious ^b^ | not serious | not serious | none | 2013 | SMD 0.46 (0.09 to 0.83) | ⨁⨁◯◯  low |

CI, confidence interval; RR, relative risk; MD, mean difference; SMD, standardized mean difference.

^a^ Downgrade one level for the risk of bias due to lack of blinding.

^b^ Downgrade one level for the inconsistency due to considerable heterogeneity (I^2^ > 75%).

^c^ Downgrade one level for the imprecision due to the total sample size is less than 400.

Table S4 Subgroup analyses of the effects of mobile health application-based interventions on self-care and quality of life.

| Outcome | Subgroup | No. studies | Pooled effect estimates | | Heterogeneity | | Subgroup differences | |
| --- | --- | --- | --- | --- | --- | --- | --- | --- |
|  |  |  | MD/SMD (95% CI) | *P* value | I^2^ | *P_Q_* |  |  |
| **Self-care maintenance** ^a^ | |  |  |  |  |  |  |  |
|  | *Mobile device* |  |  |  |  |  |  |  |
|  | Smartphone | 4 | 6.18(-11.66, 24.02)^†^ | 0.35 | 96% | <0.01 | Q=0.79, p=0.67 | |
|  | Mobile phone | 1 | 7.80(2.37, 13.23) ^†^ | - | - | - |  |  |
|  | Others ^c^ | 1 | 4.40(-0.80, 9.60) ^†^ | - | - | - |  |  |
|  | *Frequency* |  |  |  |  |  |  |  |
|  | Daily | 4 | 7.30(-10.11, 24.70) ^†^ | 0.27 | 97% | <0.01 | Q=0.36, p=0.55 | |
|  | Not daily | 2 | 3.99(-4.13, 12.10) | 0.10 | 0% | 0.77 |  |  |
|  | *Delivery personnel* |  |  |  |  |  |  |  |
|  | Multidisciplinary team | 2 | 6.29(-22.02, 34.60) ^†^ | 0.22 | 0% | 0.33 | Q=2.83, p=0.24 | |
|  | Not multidisciplinary team | 2 | 13.47(-91.19, 118.13) ^†^ | 0.35 | 94% | <0.01 |  |  |
|  | Not specified | 2 | -0.53(-58.14, 57.09) ^†^ | 0.93 | 91% | <0.01 |  |  |
| **Self-care confidence** ^a^ | |  |  |  |  |  |  |  |
|  | *Mobile device* |  |  |  |  |  |  |  |
|  | Smartphone | 4 | 6.82(-14.63, 28.27) ^†^ | 0.39 | 96% | <0.01 | Q=0.48, p=0.79 | |
|  | Mobile phone | 1 | 1.50(-6.61, 9.61) ^†^ | - | - | - |  |  |
|  | Others ^c^ | 1 | 3.90(-4.02, 11.82) ^†^ | - | - | - |  |  |
|  | *Frequency* |  |  |  |  |  |  |  |
|  | Daily | 5 | 5.57(-9.11, 20.25) ^†^ | 0.35 | 94% | <0.01 | Q=0.03, p=0.87 | |
|  | Not daily | 1 | 4.30(-6.25, 14.85) ^†^ | - | - | - |  |  |
|  | *Delivery personnel* |  |  |  |  |  |  |  |
|  | Multidisciplinary team | 2 | 2.54(-14.65, 19.73) ^†^ | 0.31 | 0% | 0.68 | Q=1.82, p=0.40 | |
|  | Not multidisciplinary team | 2 | 14.43(-136.25, 165.10) ^†^ | 0.44 | 96% | <0.01 |  |  |
|  | Not specified | 2 | -1.40(56.12, 53.31) ^†^ | 0.80 | 78% | 0.03 |  |  |
| **Self-care** ^b^ | |  |  |  |  |  |  |  |
|  | *Mobile device* |  |  |  |  |  |  |  |
|  | Smartphone | 2 | 0.16(-252.93, 253.25) ^†^ | 0.99 | 97% | <0.01 | Q=8.39, p=0.02 | |
|  | Mobile phone | 2 | -1.69(-6.18, 2.81) ^†^ | 0.13 | 0% | 0.80 |  |  |
|  | Others ^c^ | 2 | -3.30(-8.81, 2.20) ^†^ | 0.08 | 0% | 0.66 |  |  |
|  | *Frequency* |  |  |  |  |  |  |  |
|  | Daily | 4 | -1.75(-27.24, 23.75) ^†^ | 0.84 | 98% | <0.01 | Q=0.02, p=0.90 | |
|  | Not daily | 2 | -2.79(-15.95, 10.37) | 0.23 | 11% | 0.29 |  |  |
|  | *Delivery personnel* |  |  |  |  |  |  |  |
|  | Multidisciplinary team | 2 | 8.11(-139.54, 155.77) ^†^ | 0.61 | 92% | <0.01 | Q=1.46, p=0.23 | |
|  | Not multidisciplinary team | 4 | -6.84(-20.39, 6.72) ^†^ | 0.21 | 98% | <0.01 |  |  |
| **Quality of life** | |  |  |  |  |  |  |  |
|  | *Mobile device* |  |  |  |  |  |  |  |
|  | Smartphone | 6 | 0.38(-0.47, 1.22)^‡^ | 0.30 | 91% | <0.01 | Q=0.30, p=0.86 | |
|  | Mobile phone | 3 | 0.43(-0.12, 0.98) ^‡^ | 0.08 | 10% | 0.33 |  |  |
|  | Others ^c^ | 4 | 0.60(-0.40, 1.60) ^‡^ | 0.15 | 97% | <0.01 |  |  |
|  | *Frequency* |  |  |  |  |  |  |  |
|  | Daily | 11 | 0.47(0.02, 0.92) ^‡^ | 0.04 | 94% | <0.01 | Q=0.06, p=0.80 | |
|  | Not daily | 2 | 0.40 (-2.26, 3.05) ^‡^ | 0.31 | 20% | 0.26 |  |  |
|  | *Delivery personnel* |  |  |  |  |  |  |  |
|  | Multidisciplinary team | 4 | 0.39(0.13, 0.66) ^‡^ | 0.02 | 0% | 0.69 | Q=15.65, p<0.01 | |
|  | Not multidisciplinary team | 8 | 0.61(0.03, 1.18) ^‡^ | 0.04 | 95% | <0.01 |  |  |
|  | Not specified | 1 | -0.50(-0.94, -0.06) ^‡^ | - | - | - |  |  |

MD, mean difference; SMD, standardized mean difference.

^a^ measured using the Self-Care of Heart Failure Index (SCHFI).

^b^ measured using the European Heart Failure Self-care Behavior Scale (EHFScB).

^c^ Others included tablets and personal digital assistants (PDA).

^†^ Pooled effect estimates using MD.

^‡^ Pooled effect estimates using SMD.


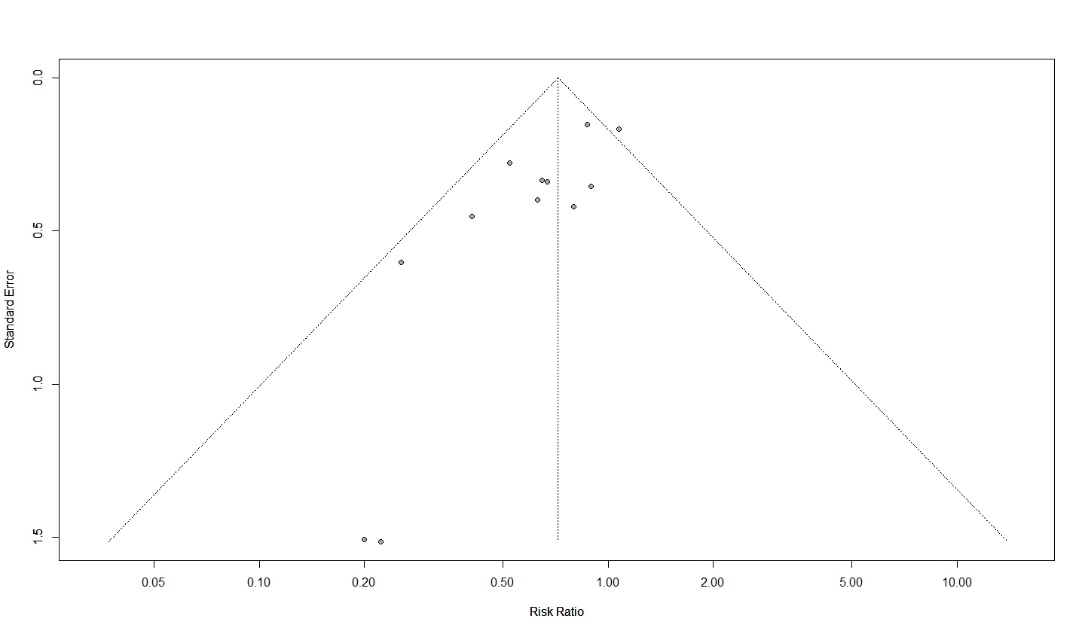


Figure S1 Funnel plots of heart failure-related hospitalization


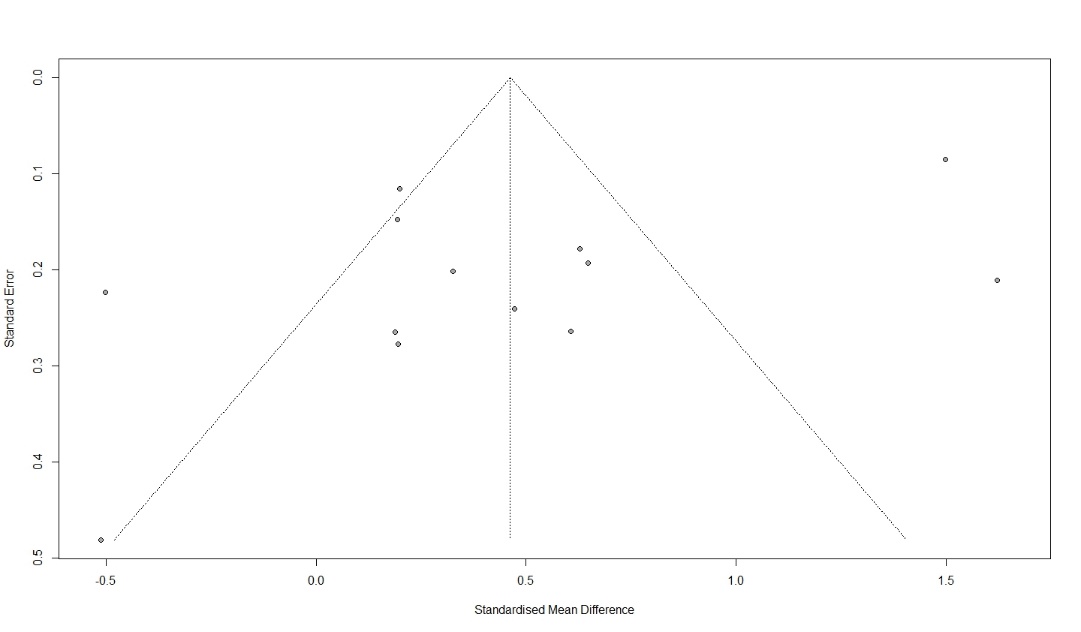


Figure S2 Funnel plots of quality of life
